# Clinical and cost-effectiveness of a New psychosocial intervention to support Independence in Dementia (NIDUS-family) for family carers and people living with dementia in their own homes: a randomised controlled trial

**DOI:** 10.1186/s13063-021-05851-z

**Published:** 2021-12-02

**Authors:** Alexandra Burton, Penny Rapaport, Marina Palomo, Kathryn Lord, Jessica Budgett, Julie Barber, Rachael Hunter, Laurie Butler, Jessica Vickerstaff, Kenneth Rockwood, Margaret Ogden, Debs Smith, Iain Lang, Gill Livingston, Briony Dow, Helen Kales, Jill Manthorpe, Kate Walters, Juanita Hoe, Vasiliki Orgeta, Quincy Samus, Claudia Cooper, on behalf of the NIDUS study team

**Affiliations:** 1 Department of Behavioural Science and Health, University College London, London, UK; 2 Division of Psychiatry, University College London, London, UK; 3 Camden and Islington NHS Foundation Trust, London, UK; 4 The Centre for Applied Dementia Studies, University of Bradford, Bradford, UK; 5 Department of Statistical Science, University College London, London, UK; 6 Research Department of Primary Care and Population Health, University College London, London, UK; 7 Faculty of Science and Engineering, Anglia Ruskin University, Cambridge, UK; 8 Division of Geriatric Medicine, Dalhousie University, Halifax, Canada; 9 Alzheimer’s Society Research Network Volunteers, London, UK; 10 College of Medicine and Health, University of Exeter, Exeter, UK; 11 National Ageing Research Institute, Royal Melbourne Hospital, Parkville, Victoria Australia; 12 Department of Psychiatry and Behavioral Sciences, UC Davis Health, University of California, California, USA; 13 NIHR Policy Research Unit in Health and Social Care Workforce, King’s College London, London, UK; 14 Division of Nursing, School of Health Sciences, City University of London, London, UK; 15 Department of Psychiatry and Behavioral Sciences, Johns Hopkins University, Baltimore, MD USA

**Keywords:** Dementia, Family carer, Psychosocial intervention, Independence

## Abstract

**Background:**

Most people living with dementia want to remain living in their own homes and are supported to do so by family carers. No interventions have consistently demonstrated improvements to people with dementia’s life quality, functioning, or other indices of living as well as possible with dementia. We have co-produced, with health and social care professionals and family carers of people with dementia, a new intervention (NIDUS-family). To our knowledge, NIDUS-family is the first manualised intervention that can be tailored to personal goals of people living with dementia and their families and is delivered by facilitators without clinical training. The intervention utilizes components of behavioural management, carer support, psychoeducation, communication and coping skills training, enablement, and environmental adaptations, with modules selected to address dyads’ selected goals. We will evaluate the effect of NIDUS-family and usual care on goal attainment, as measured by Goal Attainment Scaling (GAS) rated by family carers, compared to usual care alone at 12-month follow-up. We will also determine whether NIDUS-family and usual care is more cost-effective than usual care alone over 12 months.

**Methods:**

A randomised, two-arm, single-masked, multi-site clinical trial involving 297 people living with dementia-family carer dyads. Dyads will be randomised 2:1 to receive the NIDUS-family intervention with usual care (*n* = 199) or usual care alone (*n* = 98). The intervention group will be offered, over 1 year, via 6–8 video call or telephone sessions (or face to face if COVID-19 restrictions allow in the recruitment period) in the initial 6 months, followed by telephone follow-ups every 1–2 months to support implementation, with a trained facilitator.

**Discussion:**

Increasing the time lived at home by people living with dementia is likely to benefit lives now and in the future. Our intervention, which we adapted to include remote delivery prior to trial commencement due to the COVID-19 pandemic, aims to address barriers to living as well and as independently as possible that distress people living with dementia, exacerbate family carer(s) stress, negatively affect relationships, lead to safety risks, and frequently precipitate avoidable moves to a care home.

**Trial registration:**

International Standard Randomised Controlled Trials Number ISRCTN11425138. Registered on 7 October 2019

## Administrative information

Note: the numbers in curly brackets in this protocol refer to SPIRIT checklist item numbers. The order of the items has been modified to group similar items (see http://www.equator-network.org/reporting-guidelines/spirit-2013-statement-defining-standard-protocol-items-for-clinical-trials/).

**Table:** 

Title {1}	Clinical and cost-effectiveness of a New psychosocial intervention to support Independence in Dementia (NIDUS) for family carers and people living with dementia in their own homes: A randomised controlled trial
Trial registration {2a and 2b}.	International Standard Randomised Controlled Trials Number: ISRCTN11425138 10.1186/ISRCTN11425138
Protocol version {3}	Protocol Version 3 (06/04/2020)
Funding {4}	This work is supported by Alzheimer’s Society (UK) and is being carried out within the University College London (UCL) Alzheimer’s Society Centre of Excellence for Independence at home, NIDUS (New Interventions in Dementia Study) programme (Alzheimer’s Society Centre of Excellence grant (AS-PR2-16-002).
Author details {5a}	Department of Behavioural Science and Health, University College London, London, UK Alexandra Burton; a.burton@ucl.ac.uk Division of Psychiatry, University College London, London, UK Penny Rapaport; p.rapaport@ucl.ac.uk Jessica Budgett j.budgett@ucl.ac.uk Victoria Vickerstaff v.vickerstaff@ucl.ac.uk Claudia Cooper claudia.cooper@ucl.ac.uk Gill Livingston g.livingston@ucl.ac.uk Vasiliki Orgeta v.orgeta@ucl.ac.uk Department of Statistical Science, University College London, London, UK Julie Barber j.barber@ucl.ac.uk Research Department of Primary Care and Population Health, University College London, London, UK Rachael Hunter r.hunter@ucl.ac.uk Kate Walters k.walters@ucl.ac.uk Camden and Islington NHS Foundation Trust, London, UK Marina Palomo Marina.Palomo@candi.nhs.uk Claudia Cooper The Centre for Applied Dementia Studies, University of Bradford, Bradford, UK Kathryn Lord k.lord1@ucl.ac.uk Faculty of Science and Engineering, Anglia Ruskin University, Cambridge, UK Laurie Butler laurie.butler@anglia.ac.uk Division of Geriatric Medicine, Dalhousie University, Halifax, Canada Kenneth Rockwood Kenneth.Rockwood@Dal.Ca Alzheimer’s Society Research Network Volunteers, London, UK Margaret Ogden margaretogden@hotmail.com Debs Smith debesmith1970@gmail.com College of Medicine and Health, University of Exeter, Exeter, UK Iain Lang i.lang@exeter.ac.uk National Ageing Research Institute, Royal Melbourne Hospital, Victoria, Australia Briony Dow B.Dow@nari.edu.au Department of Psychiatry and Behavioral Sciences, UC Davis Health, University of California, California, USA Helen Kales hckales@ucdavis.edu NIHR Policy Research Unit in Health and Social Care Workforce, King’s College London, London, UK Jill Manthorpe jill.manthorpe@kcl.ac.uk Division of Nursing, School of Health Sciences, City University of London, London, UK Juanita Hoe Juanita.Hoe@city.ac.uk Department of Psychiatry and Behavioral Sciences, Johns Hopkins University, Baltimore, Maryland, USA Quincy Samus qmiles@jhmi.edu
Name and contact information for the trial sponsor {5b}	Joint Research Office, UCL Gower Street, London WC1E 6BT Email: uclh.randd@nhs.net
Role of sponsor {5c}	Neither the study sponsor (UCL Joint Research Office), nor the funder (Alzheimer’s Society) have been involved in study design; collection, management, analysis, and interpretation of data; writing of the report; or the decision to submit the report for publication. They do not have ultimate authority over any of these activities.

## Introduction

### Background and rationale {6a}

Most people with dementia want to remain living in their own homes and are supported to do so by family carers. In the UK, 61% of people with dementia live in the community [1]. Unfortunately, care at home often breaks down, necessitating a move to a care home, which can be sudden. Challenging or distressing behaviours leading to family carer stress, challenging relationships with home care services, poor self-care, and home safety risks are common reasons for this [2].

NHS England’s Well Pathway for Dementia and other national initiatives stress the importance of promoting independence for people living with dementia [3]. Living well with dementia has been conceptualised as living with quality of life, choice, autonomy, dignity, and as independently as possible. To our knowledge, only two interventions have demonstrated efficacy in enabling people with dementia to remain in their own homes for longer, to date. Both were delivered in the USA, by clinically trained staff. They were individually tailored and goal focussed [4, 5].

In stream one of the NIDUS study, we carried out qualitative interviews [6, 7] and worked with our public and patient involvement (PPI) co-production group and practitioners, to develop the theoretical model underpinning our intervention [8] to support people with dementia to attain goals they and their family carers considered critical to living as well as possible at home. The intervention is fully manualised and modular, so it can be tailored to the needs of each dyad. Modules draw on behavioural management techniques, enablement strategies, communication, carer support, and psychoeducation strategies, with material selected from existing interventions [9–12] and newly developed by the coproduction group. We tested the feasibility of the intervention with 14 people living with dementia/family carer dyads. We found it to be acceptable to participants, and we used these findings to modify the training and intervention booklets in preparation for the full trial, based on feedback from participants receiving the intervention and the researchers delivering it [13].

Our NIDUS-family intervention is delivered to people with dementia and family carers by NIDUS facilitators (graduate psychologists/social researchers with relevant experience but without formal clinical training) with supervision, training, and clinical oversight, so it can be widely implemented and give value if effective.

We originally planned to deliver the NIDUS-family intervention face-to-face to people in their homes; however, just before the trial started, lockdown restrictions due to the COVID-19 pandemic were imposed in the UK. Following discussion with our PPI members and project management team, we adapted delivery to online video or telephone sessions, so participants could continue to receive it until it is deemed safe for researchers to meet with participants face-to-face. We also adapted our intervention content, specifically to signpost to remote rather than face-to-face services, to acknowledge the restricted options for outdoor activity and the stresses of living through the pandemic lockdown. Most psychological interventions (NHS and research) for people living with dementia are being delivered remotely at the time of writing due to the needs for social distancing during the pandemic. This has illustrated the possibilities of remote delivery as well as challenges. Interventions can be potentially delivered in people’s homes very cost-effectively through video calling or over the telephone, and catchment areas can be widened. Not everyone has access to video calling, however, and engaging dyads over telephone alone may be particularly challenging.

Eight-five percent of people asked by the Alzheimer’s Society said they would want to stay at home for as long as possible if diagnosed with dementia [14]. The purpose of our intervention, to increase the time lived at home, and life quality of people living with dementia by supporting them and their family carers, is all the more pertinent in the current climate, with provision of support services diminished by the COVID-19 restrictions [15]. We have selected Goal Attainment Scaling (GAS) as our primary outcome, asking dyads to set goals that reflected what they considered most important in enabling them to live as well and as independently as possible at home. GAS can detect individualised, clinically meaningful outcomes [16], through measuring the extent to which individual patients or their families meet goals that they set. It has been used across many types of interventions and several conditions with heterogeneous outcomes, including dementia [17–19], where it has been the primary outcome in clinical trials [20, 21].

### Objectives {7}

The primary objective of the trial is to evaluate the effect of NIDUS-family and usual care on goal attainment as measured by Goal Attainment Scaling (GAS) rated by family carers, compared to usual care alone, at 12-month follow-up.

The secondary objectives are to evaluate the effect of NIDUS-family and usual care compared to usual care at 12 month follow-up on:

- Researcher rated Goal Attainment Scaling (GAS) scores [21, 22]
- Functional independence (basic and instrumental activities of daily living) as measured by the Disability Assessment for Dementia Scale (DADS) [23].
- Quality of life of the person living with dementia as measured by the Dementia Quality of Life Scale (DEMQOL) and/or the DEMQOL proxy completed by the family carer [24];
- Neuropsychiatric symptoms as measured by the Neuropsychiatric Inventory (NPI) [25];
- Family carer quality of life as measured by the CarerQol [26];
- Apathy in the person living with dementia as measured by the brief Dimensional Apathy Scale (b-DAS) [27];
- Family carer anxiety and depression as measured by the Hospital Anxiety and Depression Scale (HADS) [28];
- Potentially abusive behaviours of family carers as measured by the Modified Conflict Tactics Scale [29];
- Service use and care costs assessed using the Client Services Receipt Inventory (CSRI) [30];
- Duration of time living at home (time spent living at home during the study, up to the point of, if they occur, hospitalisation without return home, move to a care home or death).

### Trial design {8}

The trial is a two-armed, parallel group, single masked, multi-site, superiority randomised controlled trial. Randomisation is blocked and stratified by site using a 2:1 allocation ratio (two dyads are randomised to the intervention group for every one dyad randomised to the usual care group).

## Methods: participants, interventions, and outcomes

### Study setting {9}

We are recruiting potential participants via clinicians and research nurses working in NHS Trust memory clinics/older adult mental health services and GP practices based in London, Bradford, Leeds, Hull, Oxfordshire, Buckinghamshire, Kent, Essex, and Surrey and directly via the recruitment database Join Dementia Research and Twitter. Subsequent to protocol amendments made in response to COVID-19 and before study commencement, informed consent, outcome assessments, and intervention delivery are conducted via telephone or video call, depending on individual participant preference; prior to COVID-19 restrictions, we expected that outcome assessments and intervention sessions would be face to face, and if restrictions are lifted and it is safe to do so during our recruitment period (planned: April 2020 to February 2022), we will also offer this option to participants. At time of writing (October 2020), all trial processes have been conducted remotely. A list of study sites can be found here: https://www.ucl.ac.uk/psychiatry/research/mental-health-neuroscience-department/nidus/about-nidus-study/stream-2-nidus-family.

### Eligibility criteria {10}

People living with dementia/family carer dyads who meet the following inclusion criteria will be recruited:

#### People living with dementia

- Documented diagnosis of dementia of any type and severity
- Adults aged 18+ (no upper age limit)
- Living in their own homes (including sheltered accommodation, where staff are not on site 24 h a day): alone or with others
- Have a family carer/friend willing to participate in the study who is in regular (at least weekly face-to-face or telephone) contact with them;

#### Family carers/friends

- Family member or friend of the person living with dementia
- Have at least weekly face-to-face or telephone contact with the person living with dementia (not necessarily living with them)
- English speaking

People will be excluded from participation in the study if they meet any of the following criteria:

#### People living with dementia

- Receiving palliative care support and considered to be in the last 6 months of their life
- Currently enrolled in another intervention trial/research study

#### Family carers

- Lack capacity to consent
- Currently enrolled in another intervention trial/research study
- Unable to identify three goals that support living well/independence at home and within the intervention remit at baseline

### Who will take informed consent? {26a}

Trained researchers will assess capacity to consent. They will obtain verbally-recorded or written informed consent (by post after phone or video call discussion; or face to face if COVID-19 restrictions allow) from each family carer and person living with dementia prior to participation, following adequate explanation of the aims, methods, anticipated benefits, and potential hazards and burdens of the trial. Consent will be sought at least 24 h after potential participants have been given the study documentation. The researcher will explain that participants are under no obligation to enter the trial and that they can withdraw at any time during it, without having to give a reason. Family carers of people who lack capacity to consent will be asked to complete a consultee declaration form on behalf of their relative with dementia.

### Additional consent provisions for collection and use of participant data and biological specimens {26b}

We will not collect biological specimens from participants, so this section does not apply.

### Interventions

#### Explanation for the choice of comparators {6b}

Participants randomised to the control group will continue to receive usual care from NHS memory services, GP practices, and any other health and social care services during the trial period. The Quality and Outcomes Framework (QOF) recommends the development of dementia care plans that address physical and mental health, communication arrangements with secondary care, and identification of a carer in primary care and a face-to-face review every year; in practice, around half of people living with dementia in the UK have a documented annual review [31].

#### Intervention description {11a}

The intervention is delivered over a 1 year period: 6–8 manualised sessions within the first 6 months, by video call/telephone (with potential for face to face when COVID-19 restrictions permit), followed by catch-up calls every 1–2 months (at preference of participating dyad) to review progress towards goals and troubleshoot any difficulties for the remainder of the intervention period. The manualised sessions are tailored to participant’s preferences and needs for staying independent at home. In session one, the facilitator explores the participant’s identified goals and maps them to a menu of training modules, based on their priorities and concerns around maintaining independence at home. They also explore their support networks and identify gaps, sign-posting participants to existing resources and services. The modules include information and strategies addressing the following areas:

- Accepting care, arranging, and planning for the future
- Communicating with people living with dementia, family, and professionals
- Managing behaviours and challenging behaviours
- Managing physical health conditions
- Exercise, activity, and mobility
- Managing low mood, anxiety, and apathy
- Carer wellbeing and support
- Safety, environment, telecare, strategies supporting functioning at home
- Relaxation and stress management strategies
- Sleep, diet, and healthy routines

Each of these modules, if selected, is completed over 1–3 sessions; so, we anticipate most dyads will have the opportunity to complete between 2 and 4 modules. At the final appointment (the 6th, 7th or 8th session depending on the needs and preference of the dyad), the facilitator brings together old and new strategies that have worked to help the participant formulate a final action plan. They then conduct telephone calls every 1–2 months to offer support and guidance on implementing strategies and to troubleshoot any problems. Sessions are delivered to family carers and people living with dementia together or to the family carer alone. This is agreed with each dyad (depending on their goals and circumstances) so that the most appropriate arrangement is made for each session for each dyad.

The University College London (UCL) and University of Bradford (UoB) employed facilitators have been trained to deliver the NIDUS-family intervention to people living with dementia at home and their family carers. The intervention delivery training is provided by members of the research team (including an old age psychiatrist (CC), clinical psychologists (MP/PR) and the trial manager (AB), and research network volunteers from the Alzheimer’s Society (MO, DS). Initial training took place over 9 days face to face between January and March 2020. Taught content included clinical skills, taught by a clinical psychologist, with most of the time spent role-playing the sessions to practice delivery and troubleshoot scenarios that might arise. Role plays were conducted within the UCL/UoB research teams, and facilitators also role played sessions with PPI representatives with experience of caring, who then gave feedback to the research team. Facilitators were signed off to deliver the first intervention session, subsequent intervention sessions, and the final intervention session using written sign off sheets completed by members of the research team delivering the training. Facilitators attend group supervision with a qualified clinical psychologist every two weeks and can access individual support between formal supervision sessions if needed.

#### Criteria for discontinuing or modifying allocated interventions {11b}

The intervention has been modified in response to feasibility testing in a previous study [13], and therefore, no further modifications to the intervention are planned. The intervention is currently being delivered online/by telephone as opposed to face-to-face in response to restrictions imposed by the COVID-19 pandemic to ensure participant and facilitator safety. The psychosocial intervention has been assessed as low risk; therefore, we have not set criteria for discontinuation; however, participants may decide to discontinue the intervention sessions or withdraw from the project at any point. If participants decline the intervention at any point, they will be asked if they would still wish to meet with the researchers completing outcome assessments. Although it will be stressed that participants can withdraw at any time without giving a reason, any assessments that have been collected to that point will be retained and contact with participants will be maintained unless researchers are told otherwise. All contacts with participants and reasons for withdrawing from the intervention or study will be documented on the study database.

#### Strategies to improve adherence to interventions {11c}

We are flexible in terms of delivery; this may enable a subject to participate who would like to manage the time commitment the intervention requires—for example by sending material in advance and holding a session of reduced length or combining two sessions into one longer session where this is preferred by the participant. Should a family carer withdraw from the intervention, we will explore the possibility of involving a second family carer, and, if they are eligible and willing, we would consent them into the trial and invite them to complete the primary outcome (GAS) and other proxy complete follow-up questionnaires, but not the follow-up questionnaires that relate to family carer wellbeing. We will record and report where these adaptations are made to support retention of participants in the trial. Facilitators will receive guidance in the form of intervention protocols and manuals. Throughout the trial, facilitators will be supervised to ensure adherence to protocol, with weekly update and monitoring meetings. Audio recordings of 20% of intervention appointments will be used to assess fidelity to the intervention manual, and details of how these will be rated are below.

#### Relevant concomitant care permitted or prohibited during the trial {11d}

All relevant concomitant care and interventions are permitted during the trial. Many of the people with dementia taking part will be taking medications for dementia symptoms and co-existing long-term conditions as well as receiving health and/or social care services. All prescribed medications and service use will be recorded as part of the CSRI data collection. Participants enrolled in other intervention research studies at the time of recruitment will not be eligible to take part in the study.

#### Provisions for post-trial care {30}

There are no arrangements to provide the intervention to participants after the trial.

### Outcomes {12}

All outcomes will be collected at baseline and 6 and 12 months.

#### Primary outcome

- The primary outcome is functioning of the person with dementia assessed using family carer rated Goal Attainment Scaling (GAS) at 12 months follow-up. GAS is valid, reliable, and responsive to change in function in people with dementia living at home up to 12 months [21, 22]. Trained researchers will work with family carers and people living with dementia to set SMART (Specific, Measurable, Achievable, Realistic and Time-bound) goals across domains including: cognition, instrumental activities of daily life/self-care, mood, behaviour, and mobility. Family carers will evaluate ‘performance’ of the person living with dementia (or carer wellbeing if the goal relates to this) on a minimum of three and maximum of five goals set at baseline, on a 5-point scale ranging from ‘much worse’ to ‘much better’ than expected. As people will have different goals and numbers of goals, a summary formula standardises the degree of goal attainment [21], analysed as a change score. We will calculate GAS for people with dementia who have died, using procedures outlined by Gordon et al. [32]. Where death was unexpected, attainment in each domain will be rated as − 2. Where death was expected (we exclude clients who are considered to be in their last 6 months of life, but circumstances can change and we follow-up clients for 1 year), goals will be rated as achieved prior to death.

#### Secondary outcomes

Researchers conducting follow-up assessments are asked to provide their own rating on Goal Attainment Scaling [22, 33].

Family carers are asked to proxy-complete:

- Disability Assessment for Dementia Scale (DADS), a standard measure of functional independence (basic and instrumental activities of daily living) [23].
- DEMQOL proxy, a widely used measure of quality of life of people with dementia [24];
- Neuropsychiatric Inventory (NPI) [25];
- CarerQol [26];
- The brief Dimensional Apathy Scale (b-DAS) [27];
- Hospital Anxiety and Depression Scale (HADS) [28];
- Modified Conflict Tactics Scale to measure potentially abusive behaviours [29]
- Client Services Receipt Inventory (CSRI) [30] including home care, hospitalisations, respite, and all-cause time to transition from home.
- People with dementia complete, if they are able to:
- Goal Attainment Scaling [21, 22], and
- DEMQoL to rate their quality of life [24]

#### Participant timeline {13}

Table 1 details the schedule and timing of assessments. T9 and T14 represent the 6- and 12-month follow-up points respectively. In the intervention group, the 6-8 main intervention sessions occur between 0 and 6 months (time point 0 and t9) and follow-up phone calls to support implementation between T9 (6 months) and T14 (12 months). Process evaluation interviews will be conducted after all the main outcomes have been completed (T15).

**Table 1:** Participant timeline through the study

	Study period																
	Enrollment	Baseline and allocation	Post allocation													Final follow-up	Qualitative interviews
Timepoint	-*t*_1_	0	*t*_1_	*t*_*2*_	*t*_3_	*t*_*4*_	*t*_5_	*t*_6_	*t*_*7*_	*t*_8_	*t*_9_	*t*_10_	*t*_11_	*t*_12_	*t*_13_	*t*_14_	*t*_15_
Enrollment																	
Eligibility checks	X																
Informed consent	X																
Allocation		X															
Intervention delivery																	
NIDUS-family Intervention sessions			X	X	X	X	X	X	X	X							
NIDUS-family telephone follow-ups											X	X	X	X	X		
Assessments																	
Participant demographics		X															
Goal Attainment Scaling (GAS)		X									X					X	
Disability Assessment for Dementia Scale (DADS)		X									X					X	
DEMQOL/DEMQOL proxy		X									X					X	
Neuropsychiatric Inventory		X									X					X	
CarerQol		X									X					X	
Brief Dimensional Apathy Scale		X									X					X	
Hospital Anxiety and Depression Scale (HADS)		X									X					X	
Client Services Receipt Inventory (CSRI)		X									X					X	
Modified Conflict Tactics Scale		X									X					X	
Acceptability of intervention (intervention group)																X	
Qualitative interviews (10% of intervention sample)																	X

### Sample size {14}

The sample size calculation is based on the primary comparison of Goal Attainment Scaling change scores at 12 months between the intervention and usual care groups. Assuming a moderate effect size of 0.5 [34], 198 intervention and 99 control subjects are required to detect such a difference at a 5% significance level (2-tailed) with 90% power. The calculation includes an inflation for clustering due to therapist in the intervention arm (intracluster correlation coefficient (ICC) = 0.05 average cluster size = 20) and for up to 15% loss to follow-up [35]. The calculation of sample size was carried out using STATA version 14.

### Recruitment {15}

Researchers will recruit 297 family carer-people with dementia dyads from memory services or older adult mental health services in NHS Trusts and GP practices supported by the Clinical Research Networks (CRNs): North Thames, Thames Valley and South Midlands, North West London, Kent, Surrey and Sussex, and Yorkshire and Humber. We will aim to recruit 10–20 dyads per trust or GP practice area.

Clinicians and research nurses are asked to contact potential participants during the recruitment period. Verbal permission will be sought from the person living with dementia and the family carer (if present) from the identifying clinician/research nurse, and they will contact the researcher either by telephone or secure email to inform them of any interested participants. The researcher will then contact the person living with dementia and their family carer at least 24 h after the participant information sheet (PIS) has been given by the clinician/research nurse in person, 48 h if it has been sent via email, or at least 72 h if it has been sent by post.

A mail out will also be conducted to all potentially eligible participants at participating GP practices. The mail out will contain the relevant PIS and a letter asking the family carer or person with dementia to indicate their interest in participating in the study either by calling a researcher or returning a reply slip. As researchers receive responses, they will contact the person with dementia and their family carer to answer any questions they may have, check eligibility, determine the context of the situation and relationship, and then, if they would like to, make an appointment to elicit informed consent and conduct the baseline assessment.

The study is also advertised through the social media platform Twitter, the NIDUS study website, and the research organisation Join Dementia Research.

### Assignment of interventions: allocation

#### Sequence generation {16a}

Allocations will be obtained through a remote web based system: www.sealedenvelope.com provided by PRIMENT Clinical Trials Unit (CTU) and following PRIMENT Standard Operating Procedures (SOP). The randomisation list will be blocked and stratified by site using a 2:1 allocation ratio (two dyads will be randomised to the intervention group for every one dyad randomised to the control). The 2:1 ratio was selected to help enhance recruitment as more people will receive the intervention than not, and because the intervention is modular and highly individualised. By randomising more people to receive it, we will obtain a greater understanding of the intervention and the types of modules selected.

#### Concealment mechanism {16b}

Randomisation status is concealed on the remote web based system: www.sealedenvelope.com from the researchers conducting outcome assessments. Only the trial manager and the researcher assigned to deliver the intervention are aware of the participant’s allocation status.

#### Implementation {16c}

Researchers will enrol participants and collect baseline data. They will enter the data onto the Sealed Envelope database; then, as soon as data entry is complete, they will instigate the randomisation procedure. Sealed envelope provides the allocation on participant registration.

### Assignment of interventions: masking

#### Who will be blinded {17a}

The researchers collecting outcome data are blinded to group allocations. Four-to-six researchers will work in two small teams, and if a researcher delivers the intervention from one team, a researcher from the other team will conduct the outcome assessments for their participants. Clinical supervision will be conducted with the teams separately to avoid unblinding during discussions. It will not be possible to blind trial participants or researchers delivering the intervention to group allocation, nor will it be possible to blind the statisticians analysing the data due to the 2.1 participant allocation ratio, though analyses will not commence until all data are collected and the database has been locked.

#### Procedure for unblinding if needed {17b}

Researchers conducting the outcome assessments will remain blinded to participant status throughout the study unless this is accidently disclosed by a participant during outcome assessments. The trial manager will be aware of treatment allocation status and will inform clinical members of the study team of allocation status if a serious adverse event occurs. No circumstances are envisaged whereby the researchers assessing outcomes will be purposefully unblinded.

### Data collection and management

#### Plans for assessment and collection of outcomes {18a}

Data is collected at baseline and 6- and 12-month follow-up for all participants (Table 1). Those in the intervention group are asked to complete an additional measure on acceptability of the intervention at the end of the study. Approximately 10% of participants in the intervention group will participate in an additional qualitative interview about their experiences of receiving the intervention. All researchers are fully trained to use the data collection tools. All outcome assessments will be measured using validated questionnaires (please refer to the ‘Objectives’ section of this protocol for a list of validated questionnaires that will be used in this trial).

#### Plans to promote participant retention and complete follow-up {18b}

While face to face delivery is not possible due to COVID-19 restrictions, we will offer a choice of video call options or telephone contact where participants are unable or do not wish to use video call. Participants will be offered a £20 voucher to thank them for their time after each follow-up appointment (baseline and 6 and 12 months and qualitative interview).

#### Data management {19}

Data will be collected from participants manually on trial specific paper case report forms (CRFs) which will then be entered into a web-based clinical data management system, Red Pill, provided by Sealed Envelope through PRIMENT CTU. There is an agreement in place between the sponsor (UCL) and Sealed Envelope to ensure compliance and agreement with clinical trial regulations, GDPR, and data protection laws. Range checks for data values have been built into the database to minimise data entry errors.

Around 20% of GAS assessments and intervention sessions (randomly selected) will be audio-recorded for fidelity checking, and qualitative interviews will be recorded using a password protected recorder. Files will be transferred between a professional transcription service and UCL, UoB, and Anglia Ruskin University (ARU) via a secure server hosted by the transcription service. Audio files will be password protected and stored in separate folders to the transcripts on password-protected computers at UCL, UoB, and ARU and will only be accessible to members of the research team authorised to use them.

#### Confidentiality {27}

Personal data needed to re-contact participants for follow-up assessments and intervention sessions will be held securely on password protected excel spreadsheets on password-protected computers at UCL, UoB, and ARU (all co-applicants on the study). Data will be stored electronically in folders only accessible to the research team. Data collected at baseline and follow-up will be collected on case report forms (CRFs) that will not bear the participant’s name; only the participant’s trial identification number and date of birth will be used for identification, and this will be clearly explained to the participant in the PIS; these will be held securely in locked premises accessible only to the research team. Consent forms will be held electronically on password-protected computers at UCL and UoB. Only members of the research team will have access to these forms.

Audio files from recorded intervention sessions and qualitative interviews will be transcribed and all identifiable information will be removed from the transcripts including any reference to names or places. Audio files will be deleted from the recording device immediately after the safe upload has been confirmed.

#### Plans for collection, laboratory evaluation, and storage of biological specimens for genetic or molecular analysis in this trial/future use {33}

We will not be collecting any biological specimens for this study; therefore, this section is not applicable.

### Statistical methods

Following PRIMENT SOP, the detail of statistical analyses will be described in a separate statistical analysis plan that will be agreed before final trial data are made available.

#### Statistical methods for primary and secondary outcomes {20a}

Baseline demographic and questionnaire data will be summarised by treatment group using means (with standard deviations), medians (with interquartile ranges), counts, and proportions, as appropriate, to gauge the balance in characteristics between the randomised groups.

#### Primary outcome analysis

Family carer-rated Goal Attainment Scaling (GAS) global mean scores at 12 month follow-up will be summarised for the NIDUS-family and usual care groups using means with standard deviations. The outcome will be compared between the groups using a three level mixed effects model which allows for intervention arm therapist clustering and also includes a random effect for study site. The treatment effect estimate from this model will be the adjusted difference in mean GAS score which will be reported with a 95% confidence interval and *P* value. Analyses will be carried out based on the intention to treat principle, comparing the groups as randomised regardless of adherence to the intervention.

#### Secondary outcome analysis

Analyses of the continuous secondary outcome scores will take a similar approach to the analysis described for the primary outcome; however, models for these outcomes will additionally include adjustment for the associated baseline measurement.

#### Economic evaluation

The incremental cost per quality-adjusted life year (QALY) gained with the NIDUS-family intervention compared to usual care will be calculated over the 12-month duration of the trial. The primary cost analysis will be from NHS and personal and social services perspective and include NIDUS-family intervention cost in the treatment arm and resource use costs in both arms [36]. The cost of the intervention will include the cost of training, supervision, and staff time to deliver NIDUS-family intervention costed at the relevant staff grade [37]. We will collect information on impact on family carers for a societal perspective analysis. Resource use on health and social care will be collected using an adapted version of the CSRI and will be costed using the most recent nationally published sources [37].

QALYs will be calculated based on participant and family carer responses to the DEMQOL/DEMQOL proxy using the DEMQOL-U/DEMQOL-U-proxy classification system [38]. QALYs will be calculated as the area under the curve adjusting for baseline differences.

Means and 95% confidence intervals for all key results will be reported. These will be calculated using bootstrapping, adjusting for baseline and including the same covariates as specified in the statistical analysis plan. In addition, missing data will be addressed as specified in the statistical analysis plan.

Cost-effectiveness planes and cost-effectiveness acceptability curves for a range of values of willingness-to-pay for a QALY gained will be reported using the bootstrapped results.

#### Qualitative analysis of interviews

After completion of final follow-up, a 10% sub sample of participants who received the NIDUS-family intervention will be interviewed to explore their experiences of receiving the intervention and to inform adaptations and improvements to the intervention for wider roll out. This should be sufficient to reach theoretical saturation. People will be purposively recruited with varying socio-demographic characteristics to comprise a maximum variation sample, and we will invite for interview those who did not complete the intervention as well as completers to gain differing opinions. NVivo software will be used for qualitative data analysis, and a thematic analytic approach to analysing transcripts will be taken [39]. Two researchers will systematically code the transcripts into meaningful fragments and label these initial codes. Discrepancies will be discussed and resolved with the investigators overseeing the trial. The researchers will then organise the data into preliminary themes. They will discuss the coding frames within the NIDUS team using the constant comparison method to identify similarities and differences in the data. Emerging analyses will also be discussed with the NIDUS PPI group. The process evaluation data collection will be led from the ARU site, who are not involved in data collection for the main trial, and they will also lead the analysis of the process evaluation data, to reduce risk of bias.

#### Quantitative evaluation of intervention acceptability

After completion of final (12 month) study outcomes, intervention participants will be asked to rate acceptability of the intervention on a 5-point Likert scale by responding to the statement ‘the intervention helped the person I care for’ with 1 indicating strongly disagree and 5 indicating strongly agree with space for free text feedback on their experience of receiving the intervention.

#### Fidelity analysis

To analyse fidelity of delivery of NIDUS-family, the number of appointments delivered across all intervention participants will be assessed. A random sample of 20% of appointments will be audio-recorded, transcribed, and checklists applied independently by two researchers. A mean fidelity score will be produced by dividing the number of items on the checklist identified as being delivered in the appointment, by the number of items on the checklist that should have been delivered. Thresholds used in other intervention fidelity work will be adopted [40]: where 81–100% constitutes high fidelity, 51–80% is moderate fidelity, and 50% or lower constitutes low fidelity.

#### Interim analyses {21b}

As this is a trial of a psychosocial intervention and the intervention is low risk, we do not plan interim analyses. Should the independent combined Programme Management Group/Data Monitoring and Ethics Committee (PMG/DMEC) consider it necessary, they will request this and liaise with the CTU and trial sponsor.

#### Methods for additional analyses (e.g. subgroup analyses) {20b}

The following supportive analyses will be carried out for the primary and secondary outcomes using the same modelling approaches as described in the ‘Statistical methods’ section of this protocol:

• Adjusted analyses allowing for other predefined factors related to the outcome (whether the family carer participating was living with the care recipient and whether the care recipient had capacity to give informed consent to participate (as an index of dementia severity)).

• Estimation of the treatment effect adjusting for any concerning imbalances in baseline characteristics.

Subgroup analyses will explore the primary outcome in (1) those who receive a minimum ‘dose’ of the intervention, relative to the control group and those who do not, and (2) between participants receiving the intervention predominantly by telephone, video call, and (if applicable) face to face (3).

#### Methods in analysis to handle protocol non-adherence and any statistical methods to handle missing data {20c}

The number of subjects with missing data by reason will be examined for each randomisation group (and for each outcome). Characteristics of participants with and without missing outcome data will be compared using logistic regression models (with missing yes/no as the outcome) and characteristics that predict missingness identified. In a sensitivity analysis, the treatment effect will then be re-estimated with additional adjustment for baseline predictors of missingness. Further analyses based on multiple imputation methods will be considered if appropriate. Analyses will be carried out based on the intention to treat principle.

#### Plans to give access to the full protocol, participant level-data, and statistical code {31c}

The trial protocol is available via the ISRCTN registry (ISRCTN11425138). Participant-level data will be managed by PRIMENT CTU and made available on request, based on the SOP. The statistical code will be disclosed by the senior statistician on receipt of a reasonable request.

### Oversight and monitoring

#### Composition of the coordinating centre and trial steering committee {5d}

The study will be overseen by clinical academics based at UCL Division of Psychiatry, PRIMENT CTU, a Programme Management Group (PMG), and independent trial steering committee (TSC). The PMG will include the chief investigator, co-investigators, the trial manager, trial statisticians, health economists, researchers, research network volunteers, and representatives from PRIMENT CTU. The PMG will be responsible for overseeing the trial. The TSC will be chaired by an independent clinical academic and will include an independent statistician and three research network volunteers, two PRIMENT CTU representatives alongside the CI, trial manager, and lead statistician for the study.

#### Composition of the data monitoring committee, its role and reporting structure {21a}

The DMEC is combined with the PMG, as agreed with PRIMENT CTU and the funder, because this is a low risk intervention trial.

#### Adverse event reporting and harms {22}

Any serious adverse events (SAEs) which are classified as related and unexpected will be reported to the chief investigator (CI) of the study in the first instance and the local principal investigator (PI)/participant’s GP/clinician. The TSC and PRIMENT CTU will review serious adverse events and events will be reported to the research ethics committee (REC) that approved the trial. All parties will be kept informed of serious adverse events via the trial manager. All SAEs will be recorded on the online database hosted by Sealed Envelope. The CI/PI or designated researcher will complete the SAE form and the form will be preferably emailed to PRIMENT CTU within 24 h of becoming aware of the event by the trial manager. The CI or PI will respond to any SAE queries raised by the sponsor as soon as possible.

#### Frequency and plans for auditing trial conduct {23}

The PMG will meet approximately twice a year. Its remit is to review recruitment figures, serious adverse events and substantial amendments to the protocol prior to submission to the REC. The study team will also consult the independent TSC twice a year to provide overall supervision of the trial. The TSC will review the recommendations of the PMG and, on consideration of this information, recommend any appropriate amendments/actions for the trial as necessary.

#### Plans for communicating important protocol amendments to relevant parties (e.g. trial participants, ethical committees) {25}

The trial manager will submit any protocol amendments to the sponsor for approval prior to submission to the REC. The trial manager will communicate amended and approved documentation to all researchers and research sites involved in the study and will update the trial registry with any additional or revised study information.

### Dissemination plans {31a}

We will work with our PPI group in developing our dissemination plans. Findings will be disseminated in a peer reviewed journal, at an international conference, and will be presented in appropriate local forums for health and social care professionals. Participants who have indicated they are interested in the results will be sent a lay summary of the findings by post or email.

## Discussion

To our knowledge, this is the first manualised intervention tailored to personalised goals of people living with dementia and their families, delivered by facilitators without clinical training. GAS, our selected primary outcome, can detect clinically meaningful outcomes [16], through measuring the extent to which individual patients or their families meet the goals that they set. Our approach of using GAS as both an outcome measure and to directly inform a non-pharmacological intervention for people living with dementia and their families, and to train non-clinical staff to set goals, is novel.

GAS can serve as a co-intervention, where setting a goal is key to achieving it [41]. For this reason, various means of modifying the process have been developed, including standardised, protocol-specific training [42] and semi-standardised menus [43], which we will employ. Limitations of GAS include the sometimes cumbersome process of setting and adjudicating individual goals [44] and a current lack of good quality validity studies [45]. While there will be heterogeneity in goals set, this is remedied through its employment in a randomised controlled trial, with masking of who has received the intervention, and with patient assignments to treatment arms occurring only after goals have been set [33].

There have been operational issues associated with the shift to remote delivery. We worked collaboratively with Rockwood’s team to ensure the goals we set using GAS were SMART, including being achievable and relevant to the uncertain COVID and post-COVID world. A particular concern was to avoid goals where achievement would be dependent on lifting of lockdown restrictions. Researchers received additional training in delivering remote interventions and supporting participants to set up video calls. We have considered carefully how to support researchers delivering the intervention from their own homes and to ensure the boundaries of sessions are maintained in this new format; regular clinical supervision has been key to addressing this. Session content has also been adapted, as discussed in the ‘Introduction’ section. Remote delivery means that the format of our intervention is further from that delivered in our feasibility trial than originally intended. In the light of COVID-19, there have been considerable recent efforts to explore how remote and face-to-face psychological intervention delivery may differ in their impacts; one review reported minimal to no difference between telephone and face-to-face therapy in terms of the nature of the interaction between the therapist and client [46], but more information will emerge as we assimilate knowledge from the sudden shift to remote delivery in spring 2020. The usual care received by all participants will also have shifted to mainly remote delivery during lockdown, and social care receipt will have reduced. While we could not have envisaged the huge shifts to telecare the pandemic has enforced, NIDUS-family was always intended as a pragmatic intervention, comparing tailored, structured additional support sessions to usual care. The pragmatic and flexible nature of the intervention has enabled us to continue evaluating it throughout this period.

## Trial status

Protocol version number 3: 6 April 2020. The first participant dyad was recruited on 3 April 2020. Recruitment will cease in February 2022.

## Abbreviations

b-DAS: Brief Dimensional Apathy Scale
CarerQol: Care-Related Quality of Life Instrument
CI: Chief investigator
CRF: Case report form
CRN: Clinical Research Network
CSRI: Client Services Receipt Inventory
CTU: Clinical Trials Unit
DADS: Disability Assessment for Dementia Scale
DMEC: Data Monitoring and Ethics Committee
GAS: Goal Attainment Scaling
HADS: Hospital Anxiety and Depression Scale
ICC: Intracluster correlation coefficient
ISRCTN: International Standard Randomised Controlled Trials Number
NIDUS: New Interventions for Independence in Dementia Study
PI: Principal investigator
PIS: Participant information sheet
PMG: Programme Management Group
PPI: Patient and public involvement
QALY: Quality-adjusted life year
REC: Research Ethics Committee
SAE: Serious adverse event
SMART: Specific, Measurable, Achievable, Realistic and Timely
STATA: Software for Statistics and Data Science
TSC: Trial steering committee
UCL: University College London
UoB: University of Bradford
