# Correction: Clinical and cost-effectiveness of a New psychosocial intervention to support Independence in Dementia (NIDUS-family) for family carers and people living with dementia in their own homes: a randomised controlled trial

**DOI:** 10.1186/s13063-023-07768-1

**Published:** 2024-01-02

**Authors:** Alexandra Burton, Penny Rapaport, Marina Palomo, Kathryn Lord, Jessica Budgett, Julie Barber, Rachael Hunter, Laurie Butler, Victoria Vickerstaff, Kenneth Rockwood, Margaret Ogden, Debs Smith, Iain Lang, Gill Livingston, Briony Dow, Helen Kales, Jill Manthorpe, Kate Walters, Juanita Hoe, Vasiliki Orgeta, Quincy Samus, Claudia Cooper, the NIDUS study team

**Affiliations:** 1 Department of Behavioural Science and Health, University College London, London, UK; 2 Division of Psychiatry, University College London, London, UK; 3 Camden and Islington NHS Foundation Trust, London, UK; 4 The Centre for Applied Dementia Studies, University of Bradford, Bradford, UK; 5 Department of Statistical Science, University College London, London, UK; 6 Research Department of Primary Care and Population Health, University College London, London, UK; 7 Faculty of Science and Engineering, Anglia Ruskin University, Cambridge, UK; 8 Division of Geriatric Medicine, Dalhousie University, Halifax, Canada; 9 Alzheimer’s Society Research Network Volunteers, London, UK; 10 College of Medicine and Health, University of Exeter, Exeter, UK; 11 National Ageing Research Institute, Royal Melbourne Hospital, Parkville, VIC Australia; 12 Department of Psychiatry and Behavioral Sciences, UC Davis Health, University of California, California, USA; 13 NIHR Policy Research Unit in Health and Social Care Workforce, King’s College London, London, UK; 14 Division of Nursing, School of Health Sciences, City University of London, London, UK; 15 Department of Psychiatry and Behavioral Sciences, Johns Hopkins University, Baltimore, MD USA

**Correction: BMC Trials 22, 865 (2023)**

**https://doi.org/10.1186/s13063-021-05851-z**

Following publication of the original article [1], we have been notified that the name of the 9^th^ author is Victoria Vickerstaff, and not Jessica Vickerstaff. Also, their affiliation is “Research Department of Primary Care and Population Health, University College London, London, UK” instead of the originally published “Division of Psychiatry, University College London, London, UK”.

The original article has been corrected.
